# Conservation of *Salmonella* Infection Mechanisms in Plants and Animals

**DOI:** 10.1371/journal.pone.0024112

**Published:** 2011-09-06

**Authors:** Adam Schikora, Isabelle Virlogeux-Payant, Eduardo Bueso, Ana V. Garcia, Theodora Nilau, Amélie Charrier, Sandra Pelletier, Pierrette Menanteau, Manuela Baccarini, Philippe Velge, Heribert Hirt

**Affiliations:** 1 URGV Plant Genomics, INRA/University of Evry, Evry, France; 2 INRA de Tours, UR1282 Infectiologie Animale et Santé Publique, Nouzilly, France; 3 Max F. Perutz Laboratories, University of Vienna, Vienna, Austria; University of Osnabrueck, Germany

## Abstract

*Salmonella* virulence in animals depends on effectors injected by Type III Secretion Systems (T3SSs). In this report we demonstrate that *Salmonella* mutants that are unable to deliver effectors are also compromised in infection of *Arabidopsis thaliana* plants. Transcriptome analysis revealed that in contrast to wild type bacteria, T3SS mutants of *Salmonella* are compromised in suppressing highly conserved *Arabidopsis* genes that play a prominent role during *Salmonella* infection of animals. We also found that *Salmonella* originating from infected plants are equally virulent for human cells and mice. These results indicate a high degree of conservation in the defense and infection mechanism of animal and plant hosts during *Salmonella* infection.

## Introduction

Different serotypes of *Salmonella*, together with *Escherichia coli* O157:H7 and *Campylobacter spp*. are the most prominent causes of food poisoning worldwide [1]. Disease develops after consumption of contaminated food and the severity of symptoms correlates with the ability of *Salmonella* to enter epithelial cells of the intestine [2]. Although a typical infection leads to self-limiting gastroenteritis, in susceptible hosts *Salmonella* causes systemic infections by invading spleen, liver and other organs. Studies of the infection mechanisms in animals have shown that *Salmonella* actively remodels the host cell's physiology and architecture, and suppresses the host immune system by injecting a cocktail of effectors delivered by Type III Secretion System (T3SS). *Salmonella enterica* subsp. *enterica* ser. Typhimurium (*S.* Typhimurium) has two distinct T3SSs, T3SS-1 and T3SS-2, encoded by the *Salmonella* Pathogenicity Islands (SPI) SPI-1 and SPI-2 respectively [3], [4]. T3SS-1 secretes at least 14 proteins of which 6 were shown to interact with the host signaling cascades and the cytoskeleton. T3SS-2 secretes at least 19 *Salmonella enterica* specific effector proteins that are involved in survival and multiplication within the *Salmonella* containing vesicle (SCV) [5], [6]. Some of the effectors can be translocated by both T3SSs, reviewed in [7].

Until recently, little was known about the infection mechanisms of *Salmonella* in the plant kingdom [8]. Nonetheless, studies revealed that 25% of food poisoning outbreaks in the US could be associated with the consumption of contaminated vegetables or fruits [9]. Generally, it was believed that *Salmonella* rather survives on or in plant tissues after accidental contact with contaminated water or animal products. However, a growing body of evidence points to an active process in which *Salmonella* infects plant organs and uses them as a viable host [10], [11], [12], [13], [14], [15], [16], [17], [18], [19]. Besides *Salmonella*, other human-pathogenic bacteria can infect and cause severe diseases on different plant species. *Staphylococcus aureus*, *Klebsiella pneumoniae* and *E. coli* O157:H7 are able to proliferate on/in plants [15], [20]. Furthermore, the Gram-positive *Listeria monocytogenes* was shown to grow and persist on *Arabidopsis* plants [21], [22], [23].

Salmonellosis linked to contaminated vegetables raises the question whether similar mechanisms are used in animal and plant infection. Saggers et al. suggest that *Salmonella* actively attaches to plant tissues for successful colonization [17]. A large screen identified 20 out of 6000 *Salmonella* serotype Newport mutants with lower attachment ability to alfalfa sprouts [10]. Interestingly, some of the identified genes also play central roles in the pathogenicity toward animals (*e.g. rpoS* and *agfD*). In another study, two previously uncharacterized genes (STM0278 and STM0650) were characterized as important factors for the infection of alfalfa sprouts, due to their essential role in biofilm formation and swarming [24]. Light-dependent chemotaxis was shown to be involved in the internalization of *Salmonella* to lettuce leaves *via* open stomata [16]. The same group reported recently that internalization of *Salmonella* depends on the host plant [14]. On the other hand, the plant immune system seems to play an important role in preventing *Salmonella* infection. *Arabidopsis npr1* mutant, which is impaired in salicylic acid (SA) signaling, or plants over-expressing the bacterial *NahG* gene encoding the SA-metabolizing enzyme salicylate hydroxylase, are more susceptible to *Salmonella* infection [15]. Additionally, mutations in either *mpk6* or *mkk3* render plants more susceptible toward *Salmonella*, pointing to the important role of MAPK signaling cascades [25]. Interestingly, the response to *Salmonella* seems to depend on the bacterial serotype. Bacterial strains belonging to the serogroup E_4_ (O:1, 3, 19) were reported to induce chlorosis and wilting in *Arabidopsis* [12].

In order to analyze the *Salmonella* infection mechanism of plants and animals we considered three topics. First, because different physiological conditions exist in plant tissues and in warm-blooded vertebrates, we asked whether plant-originated *Salmonella* retain their virulence toward animals. Second, we wondered whether T3SS-dependent delivery of effectors, necessary for *Salmonella* infection, is of equal importance in plants as in animals? Third, we questioned whether the plant genes that are employed in response to *Salmonella* infection are different from those of animals?

## Results

### Proliferation in plants does not alter the virulence for mammalian cells

**Figure 1:**
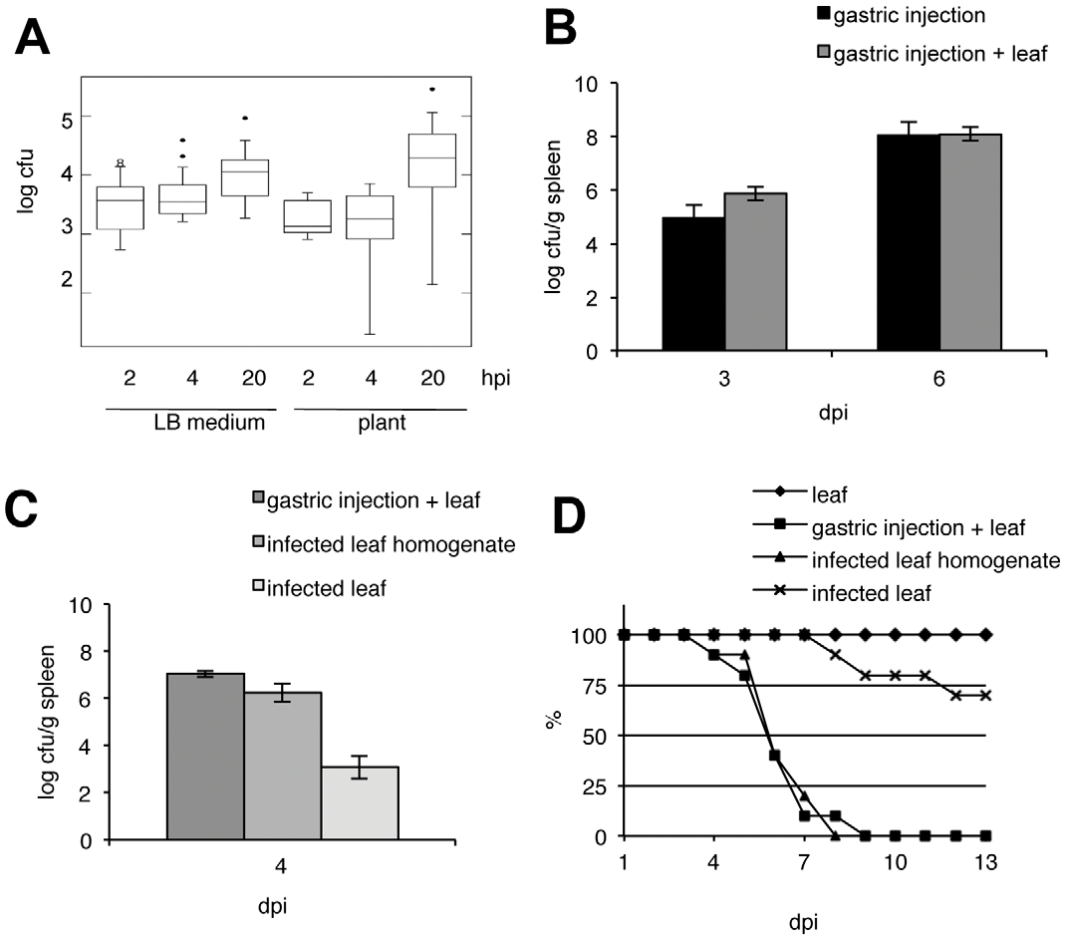
Plant-originated *Salmonella* retain their virulence toward animals. **A**; Infection and proliferation of plant-grown *S.* Typhimurium in Caco-2 epithelial cells. Caco-2 cells were infected for 1 h with bacteria originating from LB medium or *Arabidopsis* (plant), then washed and incubated for an additional 2, 4 or 20 h in the presence of gentamicin. Bacteria were harvested from lysed epithelial cells. Bacterial colony forming unit (cfu) numbers were normalized to the multiplicity of infection (moi) number; number of bacteria used for infection divided by the number of epithelial cells. Variance analysis of cfu numbers was performed using the ANOVA F-test. No differences between plant and LB grown bacteria were found (p>0.05). **B–D**; Mice infection assays. C57BL/6 mice were infected with wild-type *S.* Typhimurium 14028 s. Systemic infection in spleens was verified 3 and 6 days post infection (dpi) (**B**) or 4 dpi (**C**). **B**; Spleen infection in mice populations fed with *Arabidopsis* leaves (dark gray) is similar to infection in control population (black). **C**; Systemic infection with plant-originated *Salmonella*. Mice were infected *via* gastric injection and fed with *Arabidopsis* leaves (dark grey), fed with an infected leaves homogenate (gray) or fed with whole infected leaf (light gray). **D**; Survival of mice: fed with an infected *Arabidopsis* leaf (stars), fed with homogenate from infected leaves (triangles), infected with TSB-grown bacteria and fed with an *Arabidopsis* leaf (squares) or fed with non infected *Arabidopsis* leaf (diamonds). n = 10 mice in each group, observations were carried out for 21 dpi.

We examined the virulence potential of *Salmonella enterica* subsp. *enterica* ser. Typhimurium strain 14028s (*S.* Typhimurium) extracted from infected *Arabidopsis thaliana* leaves on the human enterocyte cell line Caco-2 [26] (ATCC HTB-37). Bacteria were collected from plants 2 days after infection and immediately used to infect Caco-2 cells with different multiplicity of infection (moi) (Supplementary Fig. S1A). The internalization of *S.* Typhimurium into Caco-2 cells (2 h time point) was similar for bacteria originating from plants or the LB medium (2×10^2^ or 5×10^2^ for plant and LB medium, respectively) (Fig. 1A). Similar colony forming unit (cfu) numbers were recovered after 4 h of incubation. However, we observed a strong increase in the number of cfu inside Caco-2 cells at 20 hours post infection (hpi): 1,7×10^3^ for LB- and 4,4×10^3^ for plant-grown bacteria. Statistical analysis of variance in the recovered populations using F-test suggests that the rates at which plant- and LB-originated bacteria proliferate in epithelial cells are comparable (p≥0.05) (Fig. 1A, Supplementary Fig. S1B).

### *Arabidopsis*-grown *Salmonella* infect mice

In order to test whether *Salmonella* bacteria proliferating inside of plants are pathogenic to animals, we infected *A. thaliana* leaves with *S.* Typhimurium and fed these leaves to C57BL/6 mice. Systemic infection was then followed by examination of the *Salmonella* cfu in spleens.

First, it was necessary to determine whether the consumption of *Arabidopsis* leaves would affect the infection process. Therefore, cfu numbers in spleens were compared between individuals, which were fed or not fed with *Arabidopsis* leaves, before oral inoculation with *Salmonella* grown on TSB medium. As shown in Figure 1B, no significant differences between these two cases were observed at 3 or 6 days post infection (dpi) (Fig. 1B).

In a second step the virulence of plant-grown *Salmonella* was assessed. For this purpose, *Salmonella* were allowed to grow *in planta* for two days before leaf discs were collected. Different variations of the infection protocol were then compared with each other: (i) mice feeding on intact non-infected leaf discs and subsequent gastric injection of TSB-grown *Salmonella* (“gastric injection+leaf” group); (ii) mice inoculated *via* gastric infection of a homogenate prepared from *Salmonella*-infected leaf discs (“infected leaf homogenate” group); or (iii) mice feeding on intact *Salmonella*-infected leaf discs (“infected leaf” group). *Salmonella* cfu in spleens were calculated at 4 dpi (Fig. 1C). All tested infection variants were successful and resulted in systemic infection. No significant differences were observed between the groups receiving gastric injection of *Salmonella* grown on TSB medium (gastric injection+ leaf group) or extracted from leaves (infected leaf homogenate group) (10^7^ and 2×10^6^ cfu/g spleen, respectively) (Fig. 1C). Similarly, feeding the mice with infected leaf discs caused systemic infection (Fig. 1C). However, the cfu recovered from the spleens of mice that were fed with intact *Salmonella*-infected leaf discs were much lower (10^3^ cfu/g spleen), indicating that bacterial infection of mice by this route is less effective (Fig. 1C, Supplementary Fig. S2).

To further analyze this system, the lethality of mice infected with *Salmonella via Arabidopsis* feeding was observed during 21 dpi (Fig. 1D). Mice fed only with uninfected *Arabidopsis* leaves (“leaf”) survived the entire 21 days of observation. In contrast, mice from the “gastric injection+leaf” or “infected leaf homogenate” groups did not survive past 9 dpi, confirming our previous observations on systemic infection. In the third group of mice fed with intact *Salmonella*-infected leaf discs (“infected leaf” group), a considerable number of animals survived 21 dpi and showed no symptoms of salmonellosis (70% survival rate) (Fig. 1D).

### *Salmonella* virulence for plants depends on T3SS-dependent delivery of effectors

**Figure 2:**
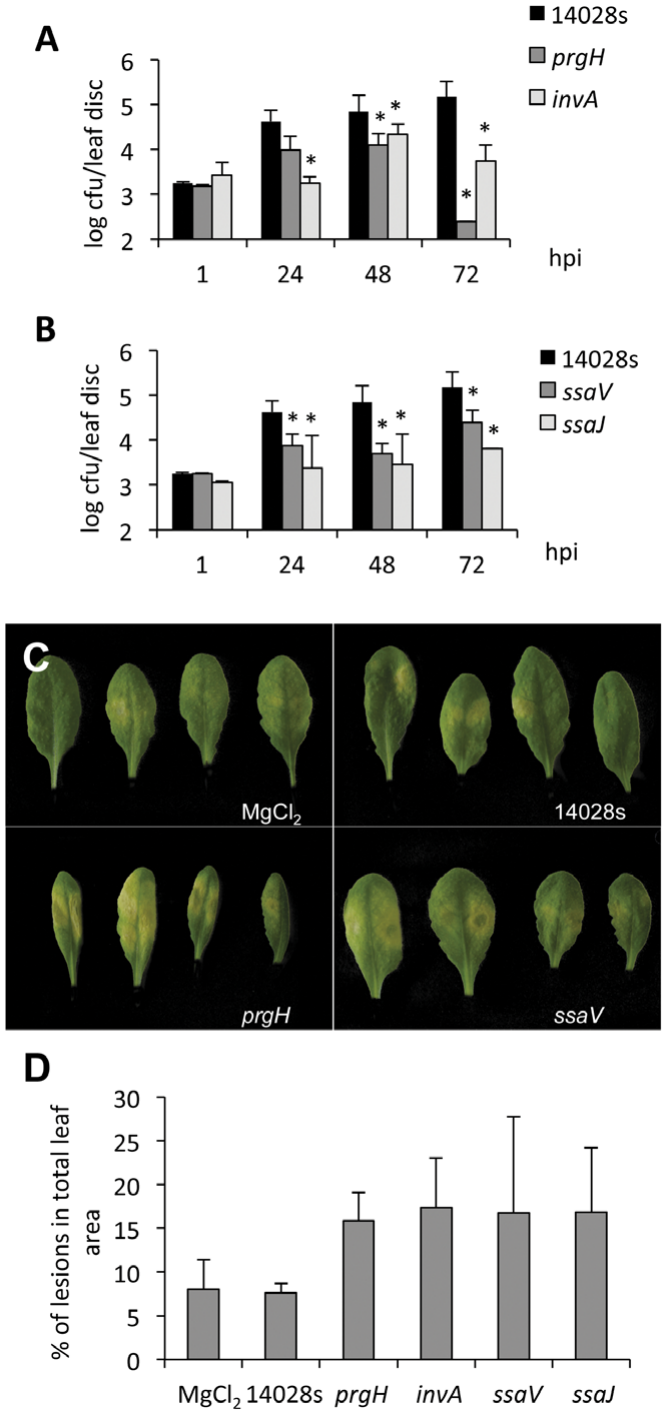
T3SS mutants of *S.* Typhimurium are less virulent for *Arabidopsis* plants. Four-week old *Arabidopsis* plants were syringe-infiltrated with bacterial solutions at OD_600_ = 0.1 of wild-type *S.* Typhimurium 14028 s or T3SS mutants. **A**; *In planta* proliferation of mutants in structural elements of SPI-1 encoded T3SS. **B**; *In planta* proliferation of mutants in structural proteins of SPI-2 encoded T3SS. Leaf discs (0.7 cm^3^) were harvested at 1–72 hours post infection (hpi). Serial dilutions were plated for cfu determination. * represents p<0.05. **C**; Symptoms on *Arabidopsis* leaves infiltrated with *Salmonella* mutants. Leaves were infiltrated with bacterial solution at OD_600_ = 0.1, photos were taken 2 days after infiltration. **D**; Quantification of symptoms provoked by infiltration with T3SS mutants or wild type 14028 s bacteria. Analysis is performed with a color-based algorithm as described in [18].

For infection of animal cells, *S.* Typhimurium uses two different T3SSs, encoded by SPI-1 and SPI-2 [5], [6], [27]. To investigate whether *Salmonella* require these secretion apparatuses to infect plants, we tested the *in planta* performance of *Salmonella* mutants that are unable to assemble a functional T3SS. In the first set of experiments, we chose *prgH^−^* (encoded by SPI-1) and *ssaV^−^* (encoded by SPI-2) isogenic mutants of virulent *S.* Typhimurium 14028s [28], [29], [30], [31]. Leaves of *A. thaliana* were syringe-infiltrated with *Salmonella*. The number of cfu was determined during 3 dpi from leaf discs of infiltrated leaves. When compared to wild type *S.* Typhimurium 14028s, both tested mutants (*prgH^−^* and *ssaV^−^*) showed reduced proliferation in plants (Fig. 2A, B). In order to verify these results, we tested an independent pair of mutants: *invA* ^−^ (encoded by SPI-1) and *ssaJ* ^−^ (encoded by SPI-2) [29], [32]. Mutants in *invA* and *ssaJ* were constructed using the Lambda-Red recombination system [33] and tested for their proliferation *in planta*. Similar to the *prgH^−^* and *ssaV* ^−^ mutants, the proliferation rate of *invA^−^* and *ssaJ^−^* in plant are lower (Fig. 2A, B). To test the possibility that the lower proliferation of bacterial mutants is a result of different multiplication rates, bacteria were grown in LB medium. No differences between the growth rates of wild type and the four tested mutants were observed (Supplementary Fig. S3A–C).

Furthermore, in addition to the lower proliferation rates observed *in planta*, symptoms caused by the *prgH^−^* and *ssaV^−^* mutants are more apparent in *Arabidopsis* plants (Fig. 2C, D). The hypersensitive response (HR) is an induced, localized cell death, which limits the spread of pathogens. HR is repressed by successful biotrophic and semi-biotrophic pathogens. Therefore, the enhanced symptoms caused by T3SS mutants may suggest the inability of *Salmonella* mutants to suppress an HR, due to the lack of an intact secretion system enabling the bacteria to inject their effector repertoire. However, activation of Mitogen-Activated Protein Kinase 6 (AtMPK6) similarly activated in response to wild type 14028 s and T3SS mutants (Supplementary Fig. S3D) suggests that HR-suppressing *Salmonella* effectors act downstream or independently of MAPK activation.

### Plants respond to *Salmonella* attack by the induction of defense genes

**Figure 3:**
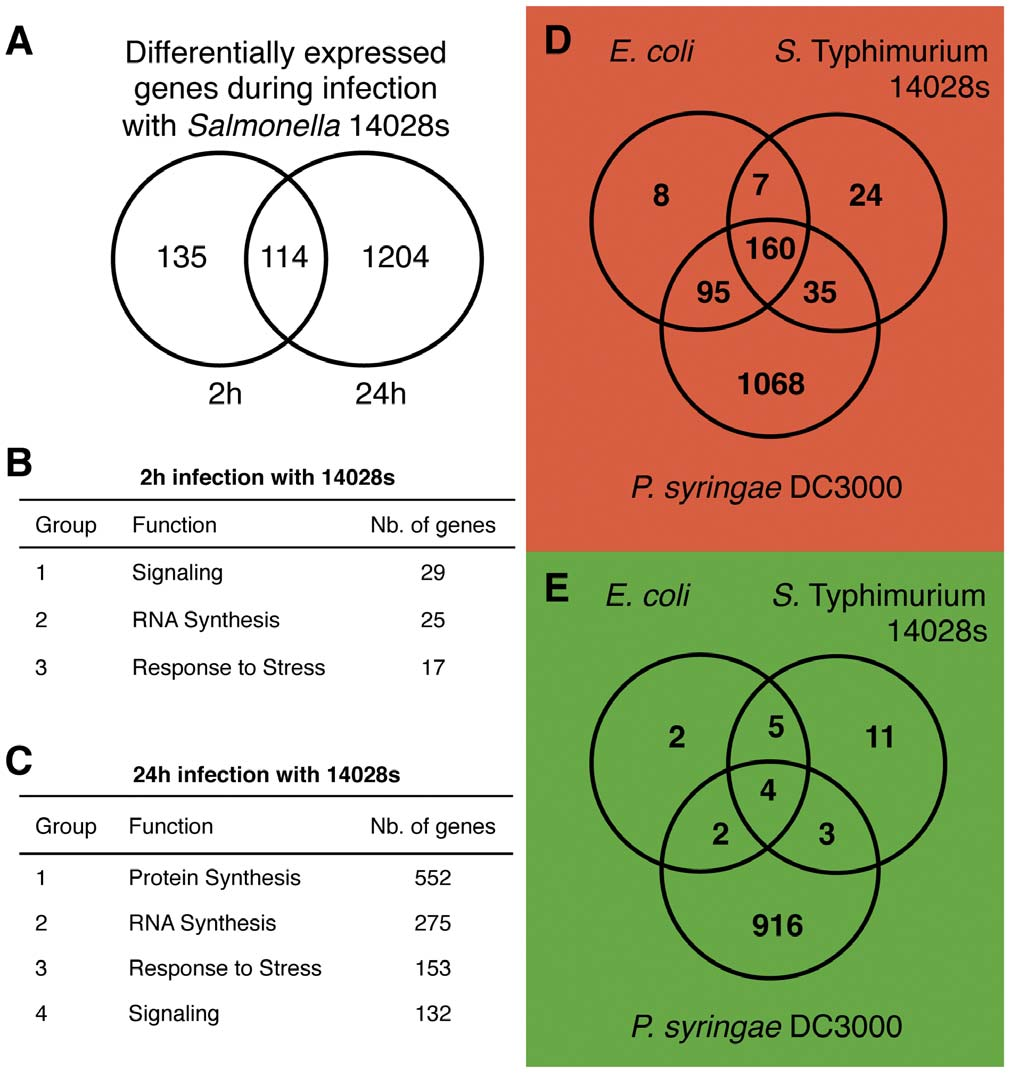
Transcriptional changes during plant infection with *S.* Typhimurium 14028 s, *E. coli* and *Pseudomonas syringae*. Fourteen-day old in vitro-grown *Arabidopsis* seedlings were placed in MS/2 liquid medium and inoculated with bacteria. Transcriptional analysis was performed at 2 or 24 h after inoculation using CATMA chips. Two biological replicates were hybridized and each hybridization was repeated with a dye-swap. Genes were considered to be differentially expressed only if they showed the same pattern in biological replicates, dye-swap and had p values<0.05. For a complete list of differentially expressed genes see Supp. Data Set S1. **A**; Overlap between genes differentially expressed at 2 and at 24 hours of infection. **B–C**; Functional GO Term groups of genes differentially expressed at 2 h (B) and at 24 h (C) after infection. **D–E**; Transcriptional analysis of plant responses to different bacteria. Fourteen-day old *Arabidopsis* seedlings were infected with *S.* Typhimurium strain 14028 s, *E. coli* DH5α or *P. syringae* DC3000 for 2 h. **D**; up-regulated genes. **E**; down-regulated genes.

To study the responses of plants to *Salmonella* attack in more detail, we performed a global transcriptome analysis of 14-day-old *A. thaliana* plants infected with *S.* Typhimurium 14028 s for 2 and 24 hours post infection (hpi). In response to *S.* Typhimurium 14028 s challenge, at 2 hpi *Arabidopsis* shows differential expression of 249 genes (Bonferroni p-value<5%), of which 226 were up- and 23 down-regulated (Fig. 3A). Among the 249 *Salmonella*-responsive genes, the largest group encodes kinases and phosphatases (24 genes), followed by genes encoding transcription factors and reactive oxygen species (ROS)-responsive proteins (18 and 16 genes, respectively) (Fig. 3B, Supplementary Data Set S1). In addition, a significant number of ubiquitin ligases and different receptors are differentially expressed upon *Salmonella* treatment (Supplementary Data Set S1). In the second step, we compared *Arabidopsis* responses to the 14028 s strain at 2 and 24 hpi. From the 249 genes differentially expressed at 2 hpi, 44% (114 genes) are similarly modified at 24 hpi (Fig. 3A). Moreover, 1204 additional genes were up- or down-regulated at this latter time point, suggesting a major transcriptional reprogramming in response to *Salmonella* infection (Fig. 3A). The function of a great number of genes differentially expressed at both time points is unknown. Among the up-regulated genes at 24 hpi with known or predicted function, the biggest group encodes for plasma membrane associated proteins (14.2%, corrected p = 2.7E-3), and the most abundant encoded protein domain is the protein kinase core domain (5.9% of up-regulated genes, corrected p = 4.6E-1). This is in line with the calculated enrichment factors (DAVID Bioinformatics Recourses 6.7 [34], [35]); the statistically most overrepresented groups are genes involved in response to different stresses and signaling (enrichment factors of 5.2 and 3.9), as well as cell wall associated genes (enrichment factor 3.86) (Fig. 3B–C). Remarkably, the biggest groups of differentially expressed genes at 24 hpi are down-regulated genes encoding for proteins involved in protein synthesis (enrichment factor of 105.46, corrected p<1E-150) and RNA synthesis (enrichment factor of 12.62, corrected p<1E-17) (Fig. 3B–C).

### Transcriptional response to *Salmonella* includes genes common in response to pathogenic and non-pathogenic bacteria, as well as a set of *Salmonella*-specific genes

To better understand the differences between the interaction of plants with *Salmonella* and other bacteria, we compared the global transcriptome response of *Arabidopsis thaliana Col*-0 upon *S.* Typhimurium 14028 s challenge, to infection with *Pseudomonas syringae* pv. *tomato* strain DC3000 (*P. syringae*) as a representative plant pathogen and *E. coli* K12 strain DH5α (*E. coli*) as a non-pathogen. The transcriptional analysis was performed on 14-day old plants at 2 hpi. As shown above, in response to *S.* Typhimurium 14028 s challenge, *Arabidopsis* shows differential expression of 249 genes (Fig. 3D, E, Supplementary Data Set S1). A total of 283 genes respond to *E. coli* (270 up- and 13 down-regulated), whereas 2283 genes responded to treatment with *P. syringae* (1358 up- and 925 down-regulated) (Fig. 3D–E). In this experimental setup, 24 genes were induced and 11 genes were down regulated specifically in response to *Salmonella* (Fig. 3D–E). These genes consist of F-box proteins, an LRR receptor and many cytoskeleton- and ER-associated proteins (Supplementary Data Set S1). The transcriptional regulation of cytoskeleton and ER proteins is particularly interesting, as it seems to reflect the physiology of *Salmonella* as an intracellular pathogen and its impact on the host cell.

A strong overlap of 164 differentially expressed plant genes is seen upon challenge with any of the three bacteria (Fig. 3D–E, Supplementary Data Set S1), probably reflecting a basic plant response to the presence of bacteria and including known pathogen-responsive genes, such as the transcription factors *WRKY22*, *WRKY33*, *WRKY40*, *WRKY48*, *WRKY53* and *bZIP5*, *bZIP60* [26], [36], [37], [38] and a number of protein kinases and phosphatases (Supplementary Data Set S1).

These findings show that *Arabidopsis* reacts to *Salmonella* attack with the induction of a large standard set of defense genes that respond similarly to pathogenic and non-pathogenic bacteria, but also that *Arabidopsis* responds to *Salmonella* infection by differential regulation of a specific group of genes.

### The *prgH^−^* mutant is unable to suppress plant immune responses

**Figure 4:**
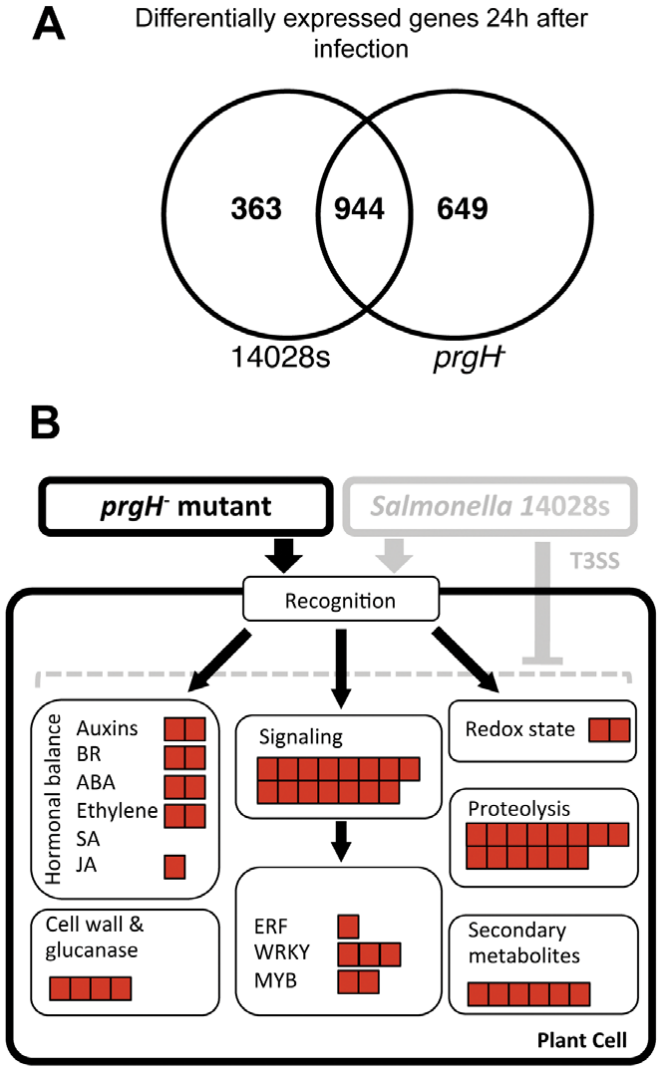
*S.* Typhimurium *prgH^−^* mutant is compromised in suppressing plant immune defenses. **A**; Comparison of transcriptional profiles of *Arabidopsis* plants after infection with 14028 s wild-type strain or the *prgH* ^−^ *Salmonella* mutant. The overlap between 14028 s- and *prgH* ^−^-regulated genes subsists of 944 genes. 649 (33% of all differentially expressed) genes are specifically regulated by *prgH* ^−^ mutant. **B**; MapMan [40] -based representation of the biggest group within the 649 *prgH* ^−^ specific genes: response to biotic stress. Graphic represents functional groups in which *prgH* ^−^ upregulated genes were identified. Each red square represent one upregulated gene in particular pathway or group. For the entire list of genes up-regulated during infection with *prgH^−^* mutant see Supp. Data Set S1.

In order to test the hypothesis, that T3SS-delivered effectors might modify the plant immune response, we analyzed the global transcriptome response of *Arabidopsis* to infection with the *Salmonella prgH* ^−^ mutant in comparison to *S.* Typhimurium 14028 s wild type, at 24 hpi. Of the more than 1300 (14028 s) or 1600 (*prgH* ^−^) differentially expressed genes, 14028 s and the *prgH* ^−^ commonly regulate a group of 944 genes (Fig. 4A). However, a group of 649 genes appears to be specifically regulated upon infection with *prgH* ^−^ (Fig. 4A, Table 1). GO term enrichment analysis (AmiGO version 1,7) [39] of these 649 *prgH* ^−^-specific genes revealed an overrepresentation of genes related to responses to biotic stress, relations with other organisms and defense mechanisms (Supplementary Data Set S1). We used the MapMan algorithm [40], which clusters differentially regulated genes to known physiological pathways or functional groups, to better characterize those genes. The majority of the *prgH* ^−^-specific genes cluster with pathways related to pathogen responses and ubiquitin-mediated protein degradation (Fig. 4B and Supplementary Fig. S4). Interestingly, this group includes *BAK1*, *BIK1*, *WRKY18* and *WRKY33*, *EIN3*, *PR4* and *PUB23*, all of which are marker genes that are up-regulated upon pathogen infections (Table 1).

**Table 1:** Differences in *Arabidopsis* gene expression levels between plants infected with *prgH^−^* mutant or wild-type 14028 s *Salmonella*.

Family	Protein	AGI	log ratio	log ratio	difference
			14028 s/mock	prgH-/mock	
PR-genes	wound-responsive	AT3G10985	1.52	2.55	**1.03**
	PR4	AT3G04720	0.25	1.32	**1.07**
	pectinesterase	AT2G45220	2.32	2.84	**0.52**
	MLO12	AT2G39200	3.09	3.42	**0.33**
	LCR78/PDF1.4	AT1G19610	1.98	3.50	**1.52**
	DNAJ heat shock	AT1G72060	2.25	3.55	**1.30**
	chitinase	AT2G43620	4.03	5.16	**1.13**
Protein binding	potease inhibitor	AT4G12500	3.33	5.18	**1.85**
	ATTI1	AT2G43510	0.85	2.39	**1.54**
	endopeptidase inhibitor	AT5G43580	2.81	3.70	**0.89**
	PGIP1	AT5G06860	1.73	2.09	**0.36**
Metabolism	AZI1	AT4G12470	2.17	3.52	**1.35**
	GH3.3	AT2G23170	1.24	2.01	**0.77**
	HR4	AT3G50480	2.23	2.91	**0.68**
	ACS7	AT4G26200	1.72	2.30	**0.58**
	PAL1	AT2G37040	0.52	0.93	**0.41**
	beta-galactosidase	AT5G56870	2.5	2.89	**0.39**
	LOX2	AT3G45140	0.61	0.72	**0.11**
Receptors	BAK1	AT4G33430	0.10	0.67	**0.57**
	BAP1	AT3G61190	0.19	0.66	**0.47**
	BIK1	AT2G39660	0.52	0.67	**0.15**
	FRK1	AT2G19190	0.60	0.70	**0.10**
Transcription factors	ATEBP	AT3G16770	1.55	2.2	**0.65**
	WRKY22	AT4G01250	1.63	2.21	**0.58**
	WRKY33	AT2G38470	0.38	0.91	**0.53**
	ERF2	AT5G47220	0.28	0.70	**0.42**
	EIN3	AT3G20770	0.51	0.82	**0.31**
	WRKY18	AT4G31800	0.57	0.77	**0.20**
Redox status	ATGSTF2	AT4G02520	1.77	3.15	**1.38**
	ATGSTF6	AT1G02930	1.73	2.86	**1.13**
	PRXR2	AT4G37520	2.86	3.45	**0.59**
	peroxidase	AT5G64100	2.64	3.17	**0.53**
	oxidoreductase	AT2G36690	2.77	3.11	**0.34**
Ubiquitin	PUB23	AT2G35930	0.56	0.91	**0.35**

All presented genes are included in the cluster 5 (Supplementary Fig. S4A). Log ratio in expression between mock treatment and infection with wild-type bacteria or log ratio in expression between mock treatment and infection with *prgH^−^* mutant were calculated using the algorithm developed for analysis of CATMA arrays [50].

A more general analysis of the transcriptional profiles shows that more than 2000 genes have higher expression levels after infection with *prgH* ^−^ than upon infection with 14028 s wild type, although the vast majority of these genes lies only close to the statistical significance level (p<0.05; Supplementary Data Set S1). Enrichment analysis (DAVID) [34], [35] of these genes classifies the up-regulated genes into cell wall, defense response and *WRKY* clusters. On the other hand, the down-regulated genes fall into the protein synthesis cluster (Supplementary Fig. S4D). Genes with the highest differences in expression levels between infection with wild type and *prgH^−^* mutant, encode for protease inhibitors, extensins, glutathione transferases, Ca^2+^-binding, wound-responsive and LRR proteins as well as chitinase and PR4 (Supplementary Tab. S2).

## Discussion

### *Salmonella* from plants retain virulence toward animals

Using epithelial cells and *in vivo* mice assays, we demonstrate in this report, that *Salmonella* originating from *Arabidopsis* leaves are as virulent as *Salmonella* grown in standard media (Fig. 1). When using *Salmonella*-infected leaf homogenates as a source for bacteria, no differences in *Salmonella* infection rates in cells or in mice were observed when compared with *Salmonella* grown *in vitro*, suggesting that proliferation *in planta* has no impact on *Salmonella* virulence for animals. Interestingly, whole leaves were clearly less infective than when *Salmonella* were derived from homogenized leaves in the feeding experiments with mice (Fig. 1C–D). Although several factors could be responsible for this phenomenon, a likely explanation is that the incomplete chewing and gastric/intestinal extraction of *Salmonella* from leaf tissues by mice may lower infection levels by hindering *Salmonella* access to the mouse intestinal epithelial target cells. These findings have important consequences for food protection and risk assessment associated with the consumption of *Salmonella*-contaminated plant products. The fact that *Salmonella* extracted from leaves show similar virulence levels to animal cells and mice as bacteria grown on standard media, suggests that *Salmonella* infected plant material possesses a considerable risk in food security and processing. The fact that *Salmonella* and other *Enterobacteriaceae* can actively use plants as hosts [41] strongly suggests that plants might represent a much larger reservoir of animal pathogens than so far estimated.

### T3SSs are required for virulence for animals and plants

Although it is accepted that *Salmonella* can infect plants and animals, it is not known whether the bacteria use different mechanisms for different hosts. Recent reports showed that *Salmonella* actively attach to plant tissues [17]. Among 20 mutants with lower attachment ability to alfalfa sprouts identified by Barak et al, some carry mutations in genes playing central roles in the pathogenicity toward animals (*e.g. rpoS* and *agfD*) [10]. Swarming and the formation of biofilms, both important factors in pathogenicity, also play a crucial role in the infection of alfalfa sprouts [24]. Previous studies showed that *Salmonella* actively access the interior of plant tissues using stomata as natural openings, but also root hairs and trichomes as points of infection [11], [14], [16], [18]. In order to answer the question whether *Salmonella* relies on intact T3SSs for infection of plants we used two different types of T3SS mutants that are unable to inject effector proteins into host cells and are therefore not virulent for animal hosts [28], [29]. Although these mutant strains had normal proliferation rates when grown in LB medium, the proliferation of these mutants in plants were strongly reduced (Fig. 2), indicating that both SPI-1- and SPI-2-encoded apparatuses are necessary to establish robust infection. Moreover, symptoms caused by these mutants were more pronounced (Fig. 2C, D), suggesting that plants can react to *Salmonella* infection with an enhanced HR rate, and/or that T3SS mutants are unable to restrain the induced HR response. Comparable results were presented very recently after tobacco infection with the wild type *S.* Typhimurium strain [19]. Wild type bacteria, but not the T3SS mutant *invA^−^*, were able to suppress the oxidative burst and the increase of extracellular pH after inoculation. Consequently the authors concluded that *Salmonella* actively suppresses plant defense mechanisms using the SPI-1 encoded T3SS [19]. Comparison of transcriptome responses to infection with *Salmonella* wild type and *prgH^−^* mutant, revealed a large number of genes with higher expression levels upon infection with the *prgH* ^−^ mutant (Fig. 4A, Supplementary Table S1, S2). The fact that this up-regulation is observed only during infection with the *prgH* ^−^ mutant but not with wild type *Salmonella* may reflect the ability of wild type bacteria to repress the plant defense machinery (Fig. 4). Although a direct search for bacterial effectors with known targets in plants like AvrPto, AvrPtoB, HopF2 or TALs, did not reveal homologous sequences in the *Salmonella* genome, such functions might be performed by yet uncharacterized effectors. Moreover, the situation may vary between plant species. Iniguez et al. (2005) showed enhanced growth of the T3SS *Salmonella spa^−^* mutant in *Medicago sativa* roots. Additionally, distinct *Salmonella enterica* serotypes might have different pathogenicity toward plants. Klerks *et al.* reported differential growth of five *S. enterica* serotypes in lettuce [42]. Interestingly, the authors pointed out that lettuce cultivars vary in their resistance/susceptibility toward *Salmonella* infection [42]. Similar results were reported by Barak et al. (2011), showing diverse resistance/susceptible phenotypes against *Salmonella* in different tomato cultivars [11]. Also internalization into plant leaves seems to be plant species-specific. Lettuce and arugula were recently reported to be easily colonized by *Salmonella*, while parsley and tomato were significantly more resistant against internalization of bacteria into their leaves [14].

Taken together, these observations suggest that *Salmonella* uses T3SS-delivered effector proteins to suppress the plant immune system. How *Salmonella* achieves the delivery of effectors across plant cell walls and plant plasma membranes remains unclear. However, numerous phytopathogenic bacteria (like *Pseudomonas*, *Erwinia* and *Xanthomonas spp.*) deliver their effectors via T3SSs, indicating that the plant cell wall is not a sufficient barrier to prevent bacteria from effector delivery [43]. T3SSs present in animal and plant pathogens are broadly conserved but show some degree of specialization in the extracellular machinery due to the adaptation to different host cell barriers. The strategy, used by *Salmonella* to overcome the mechanical and chemical barriers of plants remains to be clarified. Furthermore, the transcription of T3SSs is generally induced by specific signals coming from their animal and plant hosts and therefore another open question is how the transcriptional induction of SPI-1 and SPI-2 operons occurs during infection of host plants. The T3SS of *Pseudomonas syringae* is induced when the bacteria reaches the leaf mesophyll by plant-cell derived small and soluble compounds [44], [45]. Whether these compounds play a similar role in the induction of *Salmonella* SPIs or whether other molecules are involved, needs to be investigated in the future.

### Functional conservation of genes implicated in *Salmonella* infection of animals and plants

Our previous report and this work demonstrates that *Arabidopsis* reacts to *Salmonella* attack by inducing plant defense responses [25] (Fig. 3). Transcriptome analysis 2 h after the infection revealed 249 *Salmonella*-regulated genes of which 12% (34 genes) respond exclusively to *Salmonella*. Several of the 34 *Salmonella*-specific responsive genes encode for proteins whose functions are expected to be involved in the intracellular part of the life cycle of *Salmonella* (ER quality control, ubiquitination, ROS homeostasis and a cytoplasmic TIR-NBS-LRR protein) (Supplementary Data Set S1). BiP2 and BiP3 (up-regulated upon *Salmonella* infection) are chaperones present in the lumen of the ER and are thought to play a role in the secretion of PR proteins and the assembly of the ERF receptor [46], [47]. E3 ubiquitin ligases are essential factors in plant immunity and 6 members of this gene family are specifically up-regulated upon *Salmonella* challenge [48]. Finally, the *TIR-NBS-LRR* family includes receptors (*R*-genes) for bacterial effectors (*e.g.* RPS2) that activate effector-triggered immunity (ETI). Interestingly, 66% of *Salmonella*-responsive genes react also to treatment with *P. syringae* and *E. coli* (Fig. 3D–E). This list includes known PAMP-responsive genes as the *WRKY* transcription factors *WRKY22*, *WRKY33* and *WRKY53*. A very similar reaction was observed after infection with the pathogenic *E. coli* strain O157:H7 [49], suggesting that the regulation of these genes represents a general PAMP-triggered response to bacteria.

Overall, our results suggest that *Salmonella* employs the same T3SS machinery for delivering effectors to succumb plants as well as animals as hosts. Several plant genes that become induced upon *Salmonella* infection encode highly conserved factors that were found to play essential roles during animal infection. Future studies will reveal whether these factors play similar roles in the infection cycle of plants. The conserved nature of the *Salmonella* infection strategy and the host response mechanisms of plants and animals could underlie the ability of *Salmonella* to transit between animal and plant species and should be of more than pure academic interest to future studies.

## Materials and Methods

### Ethics statement

Animal experiments in this study were carried out in strict accordance with the French recommendations (number 2001-131 from 4.02.2001 and number 2001-464 from 29.05.2001). The protocol for this study was approved (N° CL2008-16) by the Ethics Regional Committee for Animal Research “Comité Régional d'Ethique pour l'expérimentation Animale” Centre Limousin, that is recognized by the French Ministry for Research and Education. All efforts were made to minimize suffering.

### Epithelial cells assay

Wild type *Salmonella enterica* subsp. *enterica* ser. Typhimurium strain 14028 s was grown either *in planta* for two days, or in LB medium until early logarithmic phase. Bacteria were harvested and resuspended in PBS for infection. Human Caco-2 cells [26] (ATCC number HTB-37) were infected with different multiplicity of infection (moi) for 1 h at 37°C. Cells were subsequently washed in PBS supplemented with 100 µg/mL gentamicin and incubated in growth medium (with 10 µg/mL gentamicin) for an additional 2, 4 or 20 h at 37°C. Cells were lysed in 0.5% Triton X-100 and adequate dilutions were plated on LB plates in order to monitor the intracellular *Salmonella* population. Calculations were made on the basis of cfu recovered from lysed Caco-2 cells. cfu numbers were normalized to either moi number; number of bacteria used for infection divided by the number of epithelial cells or to bacterial input population, as input we defined the number of bacteria recovered after 2 h post infection. Statistical analysis was performed using the Analysis of Variance (ANOVA) F-test. For detailed descriptions, see Supplementary Material and Methods S1.

### Mice infection assay

Eight-week-old C57BL/6 mice were orally inoculated with approximately 5×10^5^ cfu of *S.* Typhimurium 14028 s provided in four different ways; (i) animals were inoculated by gavage with 0.2 mL of an inoculum, (ii) a piece of a healthy *A. thaliana* leaf (0.6 cm^2^) was given to each animal to eat after which animals were orally inoculated as the first group of mice, (iii) a piece of a leaf infected with *S.* Typhimurium 14028 (0.6 cm^2^) was provided as food to each animal, (iv) mice were inoculated by gavage with a homogenate of infected *A. thaliana* leaves. Spleen colonization was estimated at day 3 and 6 or day 4 post-inoculation. The course of survival was recorded for 21 days following inoculation. Animal care and handling were conducted in accordance with institutional guidelines. For detailed descriptions, see Supplementary Material and Methods S1.

### Plant infection

In order to asses the *Salmonella* proliferation rate in plants, soil-grown, 4-week old *Arabidopsis thaliana Col*-0 plants were infiltrated with wild type *S.* Typhimurium strain 14028 s or *prgH^−^*, *invA^−^*, *ssaV^−^* and *ssaJ^−^* isogenic mutants, using syringe infiltration. Bacteria were grown until early log phase in LB medium, washed and resuspended in 10 mM MgCl_2_. Infiltration solution was adjusted to OD_600_ = 0.1, (1.7×10^8^ bacteria/ml). Bacterial population was monitored during 3 days post infiltration as described in [25]. The lesions were calculated as percentage of diseased regions on the base of whole leaf area using the “Salmonella Analyzer” software [18] based on color-differences detecting algorithm, 5 leaves per treatment were used for calculation, experiment was repeated 4 times.

For transcriptome analysis 14-day old *Arabidopsis thaliana* seedlings were submerged in sterile MS/2 medium without sucrose overnight at 24°C prior to bacterial treatment. *Salmonella* Typhimurium 14028 s or its *prgH^−^* mutant were grown until early log phase and washed with 10 mM MgCl_2_. Infections were performed by inoculation of the MS/2 media with bacteria at a final OD_600_ = 0.1 for 2 and 24 h. Treatments with *E. coli* strain DH5α and virulent *Pseudomonas syringae* DC3000 pathovar *tomato* (*Pst*) were performed in similar manner, for 2 hours.

### Transcriptome analysis

Total RNA was extracted from *A. thaliana Col*-0 plants; 2 or 24 hours after the infection with bacteria, amplified and fluorochemically stained as described in [50]. Two biological repetitions were performed. Hybridizations to CATMA chips [51], [52] were repeated using the dye-swap technique. Transcriptome data were analyzed using the algorithm developed for CATMA chips, as described in [50]. Expression levels were compared to a mock treatment (10 mM MgCl_2_). For detailed descriptions, see Supplementary Material and Methods S1.

### Data Deposition

Microarray data from this article were deposited at Gene Expression Omnibus (http://www.ncbi.nlm.nih.gov/geo/), accession Nb.: GSE20996, GSE23790 and GSE23791, and at CATdb (http://urgv.evry.inra.fr/CATdb/; Projects: Au07-07_Salmonella and AU10-06_Salmonella_EduardoB) according to the “Minimum Information About a Microarray Experiment” standards.

## Supporting Information

Figure S1 **A**: Multiplicity of infection (moi) used to infect Caco-2 cells. Bacteria were recovered from *Arabidopsis* leaves (squares) or LB medium (diamonds) and cfu numbers were calculated. Because of the experimental design, moi numbers (cfu number/Caco-2 cells) have been calculated only *post factum* from serial dilutions of bacterial solution used to infect epithelial cells. Bacteria recovered from plants or LB medium were used immediately. In the calculations of differences in proliferation between bacteria originated from plants and LB medium, only experiments where moi numbers were comparable between the two groups were taken into account. **B**: Infection and proliferation of plant-grown *S.* Typhimurium 14028 s in Caco-2 epithelial cells. Caco-2 cells were infected for 1 h with bacteria originating from LB or plants, then washed and incubated for an additional 2, 4 or 20 h in the presence of gentamicin (10 µg/mL). Bacteria were harvested from lysed epithelial cells and serial dilutions plated on LB agar. Bacterial cfu recovered from Caco-2 cells after 2 h incubation was used for normalization (bacterial invasion). In contrast to the normalization on moi base (Fig. 1A), this graph presents the ability of *Salmonella* to proliferate within Caco-2 cells. It is very striking that the plant-originated bacteria proliferate at a higher rate than those originated from LB medium. **C–E**: Localization of GFP-expressing *S.* Typhimurium grown in plant in the pre-nuclear region of an epithelial cell 20 h post infection. Caco-2 cells were fixed and labeled with rhodamine-phalloidin to visualize actin filaments. Observations were done using CLSM. **C**; Rhodamine channel; Ex 543, Em LP 590. **D**; GFP channel; Ex: 488, Em: BP 515–530. **E**; overlay. Arrows show GFP-expressing *Salmonella*, * nucleus.
(TIF)

Figure S2 Bacterial input used in mice infection experiments. *S.* Typhimurium 14028 s was infiltrated and allowed to grow in *Arabidopsis* leaves for two days. 0.6 cm^2^ leaf discs from infected leaves were cut off and provided as food directly (infected leaves) or pooled together (25 discs) and homogenized in PBS (leaf homogenate). Homogenate was force-fed to mice. cfu numbers present in different leaves and in homogenate were calculated on a base of serial dilutions made from discs cut out from the same leaf (infected leaves) or an aliquot of the prepared disc homogenate (leaf homogenate). Dilutions were prepared in sterile water and plated on LB agar plates.
(TIF)

Figure S3 **A**: Proliferation rates of *Salmonella* mutants in LB standard medium. 14028 s wild type and *prgH^−^*, *invA^−^*, *ssaV^−^*, *ssaJ^−^* isogenic mutants were grown in liquid LB medium at 37°C. Optical density (600 nm) was measured every 30 min. **B**: Duplication time was calculated on the base of logarithmic section of growing curves. **C**: Student's t-test results. Compared were the growing rates of the wild type 14028 s strain with growing rates of mutant strains, all strains have similar duplication times. **C**: MPK6 activity upon infection with the wild-type 14028 s Salmonella strain, or the *prgH* ^−^ and *ssaV* ^−^ mutants. Two weeks old *A. thaliana* seedlings were treated with either 10 mM MgCl_2_ or *S.* Typhimurium wild type or mutants for 20 min. Endogenous MPK6 was immunoprecipitated from total protein extraction. Myelin basic protein (MBP) was used as substrate to test the activity of MPK6 (activity). Protein amounts were detected by Western blotting with antibodies specific for AtMPK6 (MPK6).
(TIF)

Figure S4 **A**: k-means cluster analysis of the 1956 differentially expressed genes upon infection with wild type 14028 s *Salmonella* and the *prgH* ^−^ mutant. Expression levels 24 h after infection with *prgH^−^* or 14028 s wild type was calculated on the base of CATMA array hybridization. Each infection was compared independently to mock treatment (10 mM MgCl_2_). As differentially expressed genes we regarded genes with p value<5% (Bonferroni method). **B–C**: MapMan representations of the genes up regulated under infection with *prgH* ^−^ mutant in the ubiquitin-dependent protein degradation pathway. 47 genes are represented. RING and E2 ligases form the second largest cluster. Expression levels 24 h after infection with *prgH^−^* or 14028 s wild type was calculated on the base of CATMA array hybridization. Each infection was compared independently to mock treatment (10 mM MgCl_2_). Differentially expressed genes are presented in Fig. 4. The entire list of differentially expressed genes is presented in Supplementary Data Set S1. **D**: Enrichment of genes with higher expression levels 24 hours after infection with *prgH^−^* mutant compared to the response to infection with 14028 s wild type. GO Term analysis and clustering was done with the help of DAVID Bioinformatic Resources 6.7 at the National Institute of Allergy and Infectious Diseases (NIAID).
(TIF)

Data Set S1 Complete lists of genes differentially expressed after infection with bacteria. Fourteen-day old *Arabidopsis* Col-0 seedlings were placed in MS/2 medium over night and then medium was inoculated with *Salmonella* Typhimurium strain 14028 s wild-type, *E. coli* DH5α, *Pseudomonas syringae* DC3000 for 2 hours or with 14028 s *Salmonella* wild type or its *prgH* ^−^ isogenic mutant for 24 hours. Transcriptome analysis was carried out using CATMA chips as described in [50]. Two biological replicates and a dye swap repetition were used as a base for statistical analysis. Genes with the same patter in all hybridization events and with a p value≤0.05 were taken into account.
(XLS)

Table S1 AmiGO enrichment analysis of genes up regulated exclusively during infection with *prgH* ^−^ mutant (http://www.arabidopsis.org/tools/bulk/go/index.jsp).
(DOC)

Table S2 List of genes with the highest difference in expression levels between infections with *prgH^−^* mutant and wild-type *Salmonella*. Table represents log ratios between the mock treatments and the treatment with either *prgH^−^* mutant or wild-type *Salmonella*, and the difference in those two ratios (log *prgH*-log 14028 s ratio colon) calculated on the base of CATMA microarray analysis. A difference of 1 means that the expression level upon *prgH^−^* infection is 2 times higher than upon infection with 14028 s. Value of 1 = log_2_ 1 difference (2× higher expression) between *prgH^−^* and 14028 s treatments.
(DOC)

Material and Methods S1 Detailed description of epithelial cells and mice infection protocols. Description of CATMA-based transcriptome analysis performed in this study with statistical evaluation of the data.
(DOC)
