# Supplementary figures and images for: Conservation of Salmonella Infection Mechanisms in Plants and Animals
Source: PLoS One. 2011 Sep 6;6(9):e24112. doi: 10.1371/journal.pone.0024112 (PMC3167816; doi:10.1371/journal.pone.0024112)

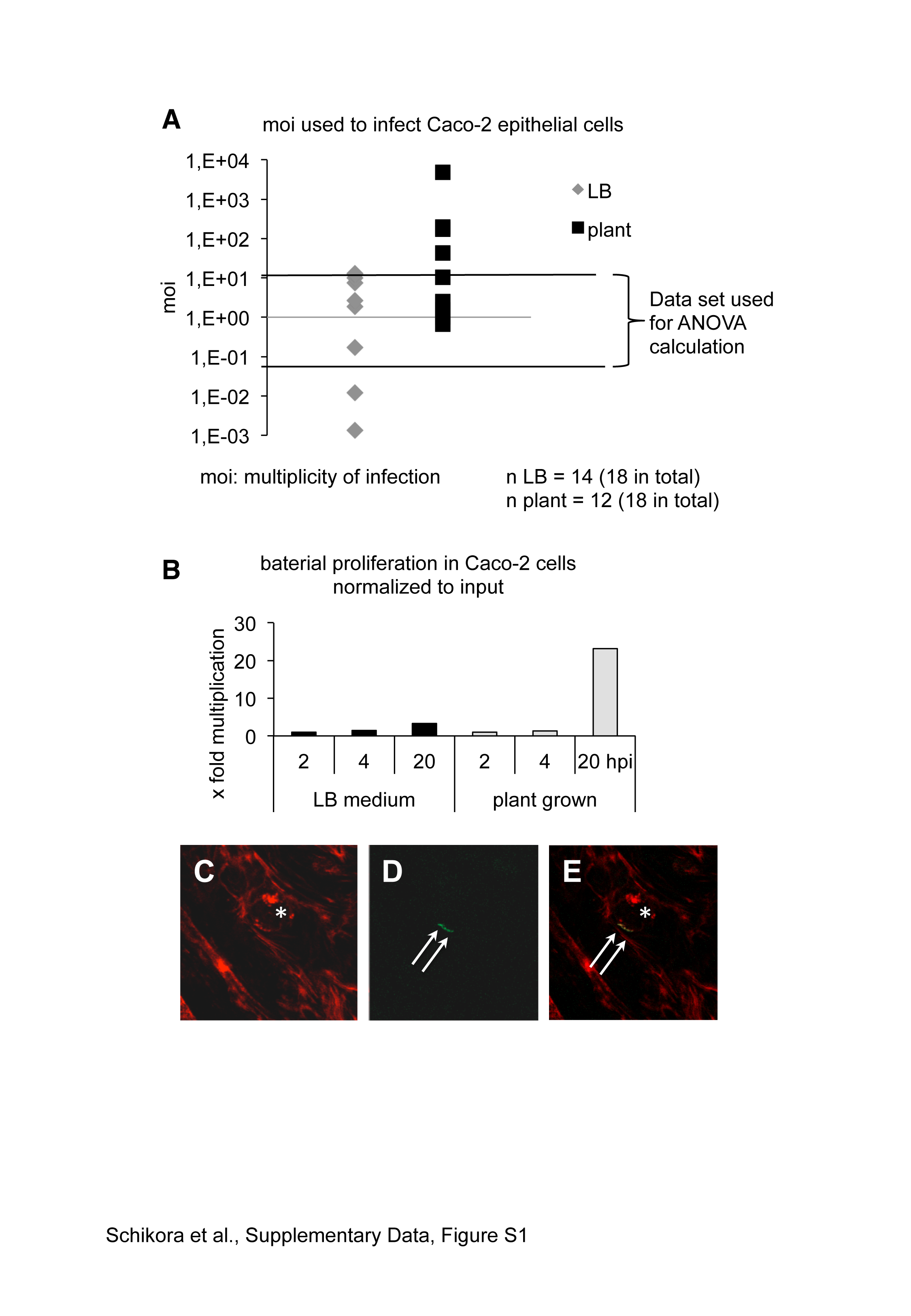

Supplement: Figure S1 — A: Multiplicity of infection (moi) used to infect Caco-2 cells. Bacteria were recovered from Arabidopsis leaves (squares) or LB medium (diamonds) and cfu numbers were calculated. Because of the experimental design, moi numbers (cfu number/Caco-2 cells) have been calculated only post factum from serial dilutions of bacterial solution used to infect epithelial cells. Bacteria recovered from plants or LB medium were used immediately. In the calculations of differences in proliferation between bacteria originated from plants and LB medium, only experiments where moi numbers were comparable between the two groups were taken into account. B: Infection and proliferation of plant-grown S. Typhimurium 14028 s in Caco-2 epithelial cells. Caco-2 cells were infected for 1 h with bacteria originating from LB or plants, then washed and incubated for an additional 2, 4 or 20 h in the presence of gentamicin (10 µg/mL). Bacteria were harvested from lysed epithelial cells and serial dilutions plated on LB agar. Bacterial cfu recovered from Caco-2 cells after 2 h incubation was used for normalization (bacterial invasion). In contrast to the normalization on moi base (Fig. 1A), this graph presents the ability of Salmonella to proliferate within Caco-2 cells. It is very striking that the plant-originated bacteria proliferate at a higher rate than those originated from LB medium. C–E: Localization of GFP-expressing S. Typhimurium grown in plant in the pre-nuclear region of an epithelial cell 20 h post infection. Caco-2 cells were fixed and labeled with rhodamine-phalloidin to visualize actin filaments. Observations were done using CLSM. C; Rhodamine channel; Ex 543, Em LP 590. D; GFP channel; Ex: 488, Em: BP 515–530. E; overlay. Arrows show GFP-expressing Salmonella, * nucleus. (TIF) [file pone.0024112.s001.tif]

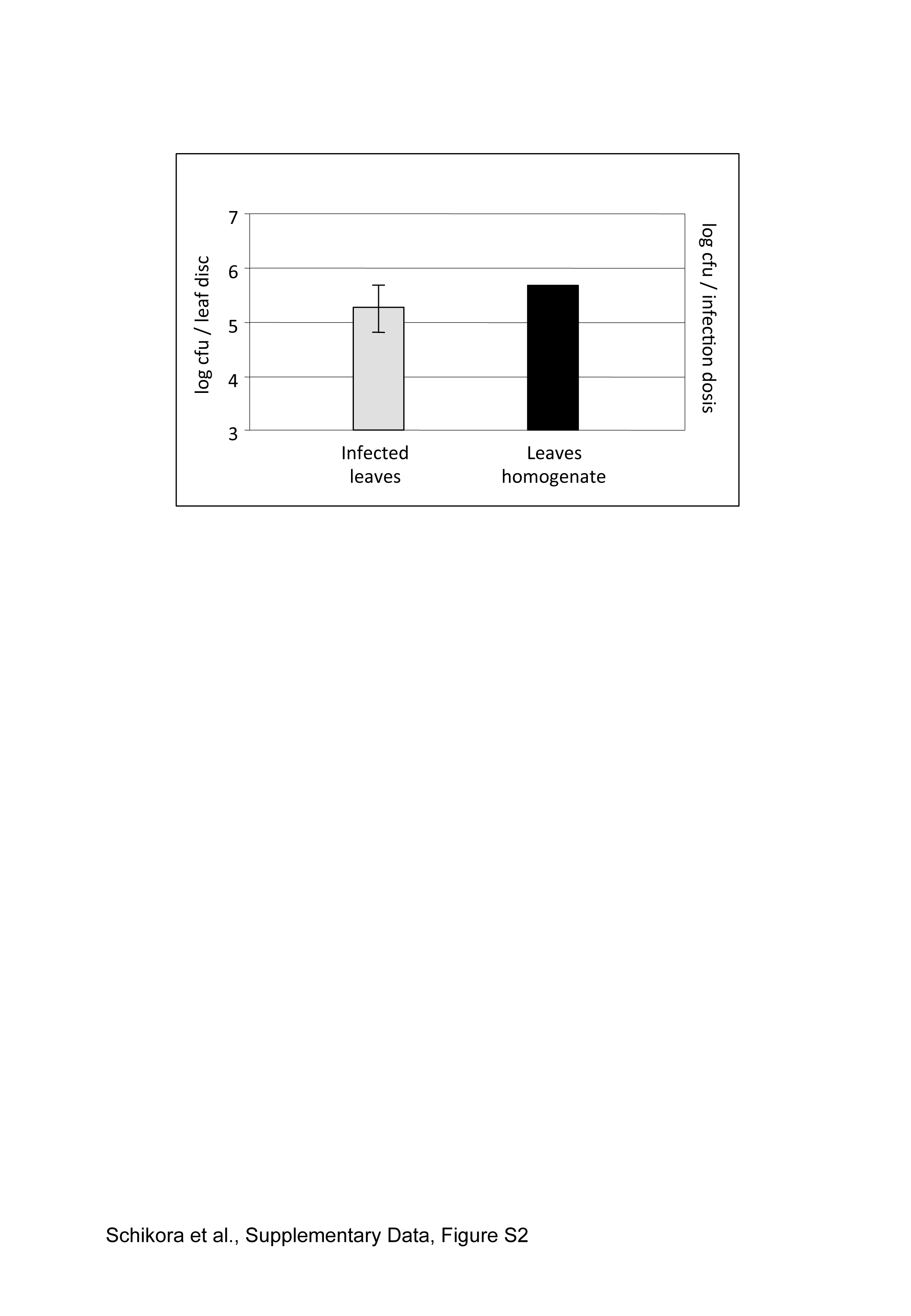

Supplement: Figure S2 — Bacterial input used in mice infection experiments. S. Typhimurium 14028 s was infiltrated and allowed to grow in Arabidopsis leaves for two days. 0.6 cm2 leaf discs from infected leaves were cut off and provided as food directly (infected leaves) or pooled together (25 discs) and homogenized in PBS (leaf homogenate). Homogenate was force-fed to mice. cfu numbers present in different leaves and in homogenate were calculated on a base of serial dilutions made from discs cut out from the same leaf (infected leaves) or an aliquot of the prepared disc homogenate (leaf homogenate). Dilutions were prepared in sterile water and plated on LB agar plates. (TIF) [file pone.0024112.s002.tif]

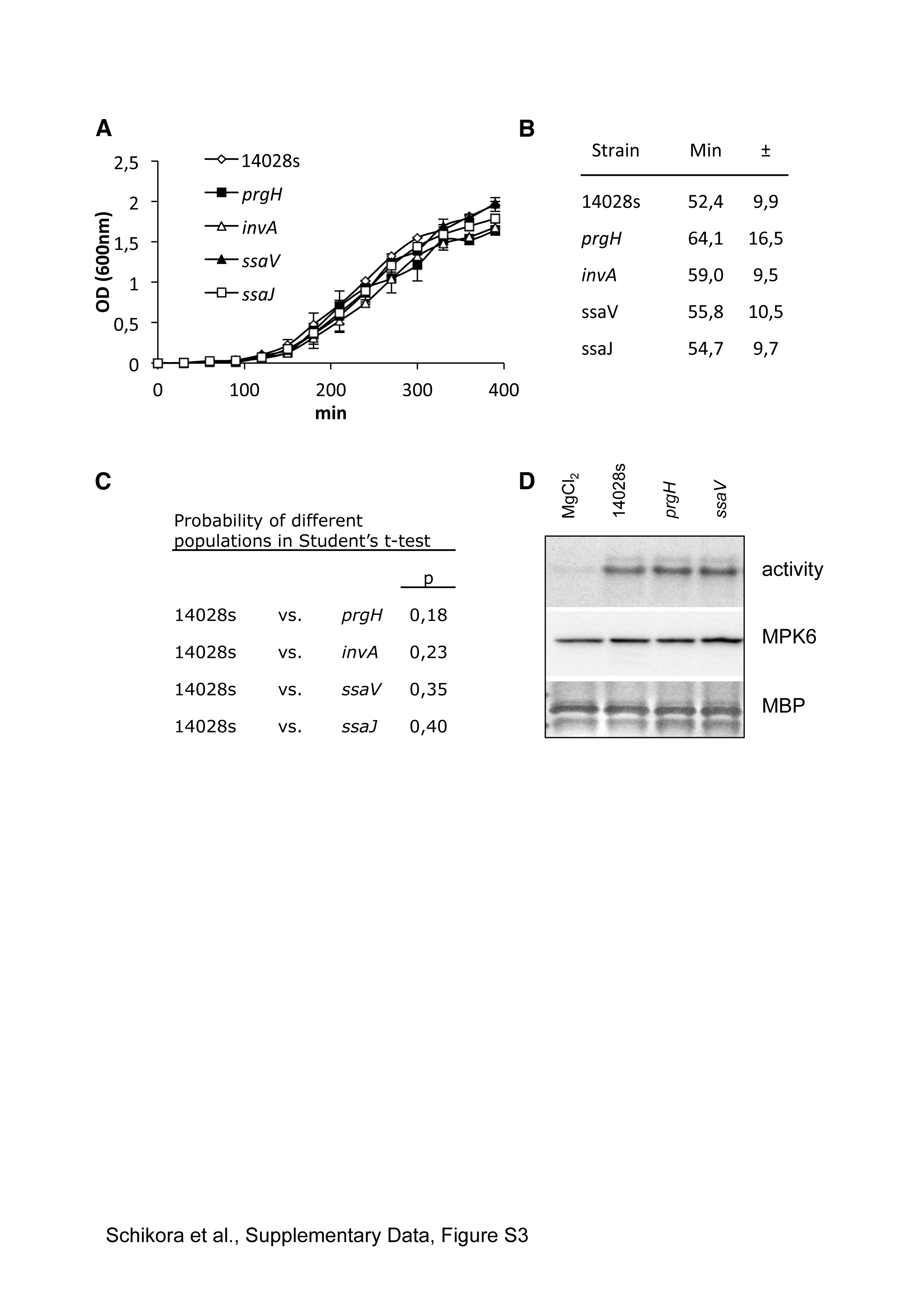

Supplement: Figure S3 — A: Proliferation rates of Salmonella mutants in LB standard medium. 14028 s wild type and prgH−, invA−, ssaV−, ssaJ− isogenic mutants were grown in liquid LB medium at 37°C. Optical density (600 nm) was measured every 30 min. B: Duplication time was calculated on the base of logarithmic section of growing curves. C: Student's t-test results. Compared were the growing rates of the wild type 14028 s strain with growing rates of mutant strains, all strains have similar duplication times. C: MPK6 activity upon infection with the wild-type 14028 s Salmonella strain, or the prgH − and ssaV − mutants. Two weeks old A. thaliana seedlings were treated with either 10 mM MgCl2 or S. Typhimurium wild type or mutants for 20 min. Endogenous MPK6 was immunoprecipitated from total protein extraction. Myelin basic protein (MBP) was used as substrate to test the activity of MPK6 (activity). Protein amounts were detected by Western blotting with antibodies specific for AtMPK6 (MPK6). (TIF) [file pone.0024112.s003.tif]

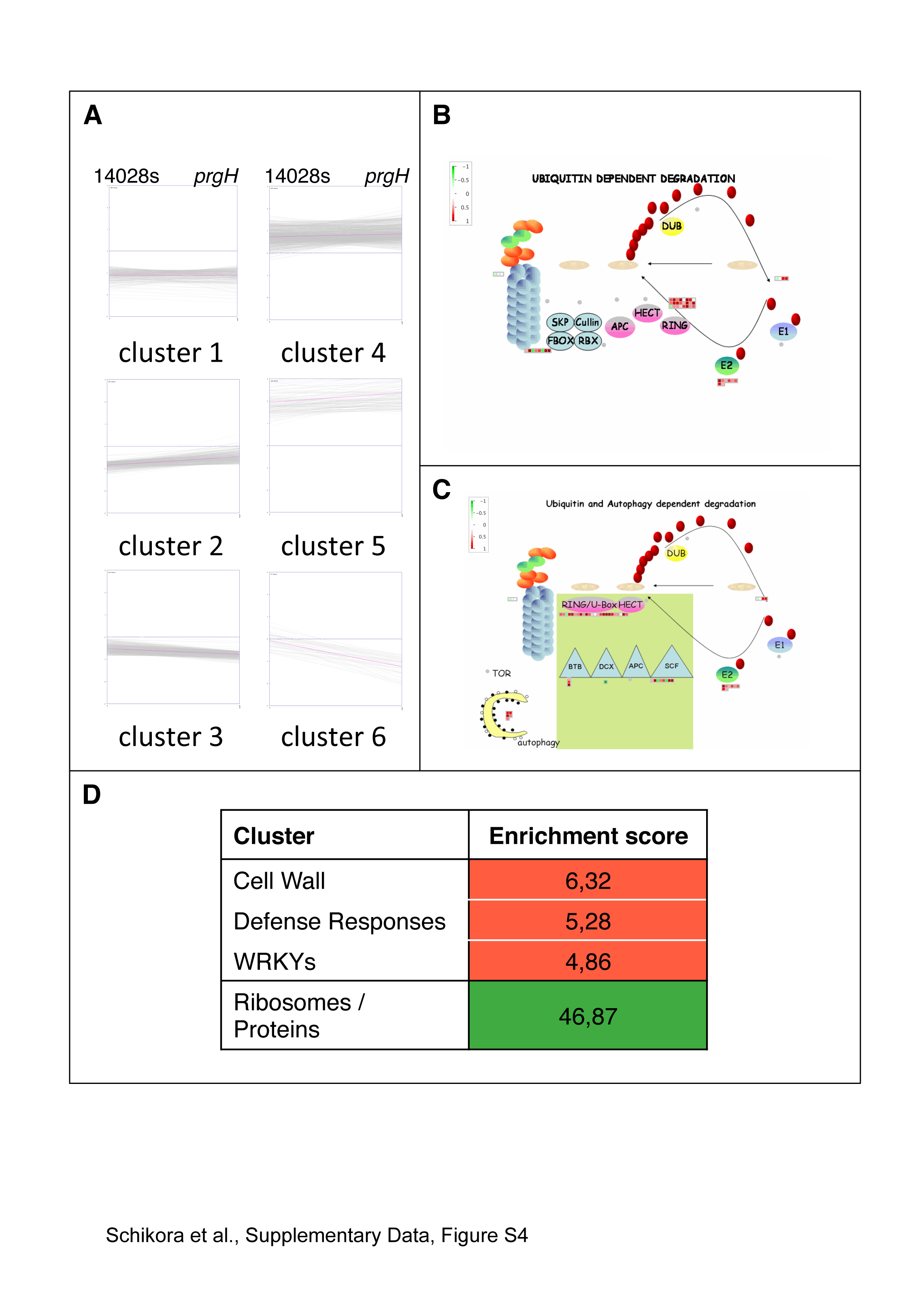

Supplement: Figure S4 — A: k-means cluster analysis of the 1956 differentially expressed genes upon infection with wild type 14028 s Salmonella and the prgH − mutant. Expression levels 24 h after infection with prgH− or 14028 s wild type was calculated on the base of CATMA array hybridization. Each infection was compared independently to mock treatment (10 mM MgCl2). As differentially expressed genes we regarded genes with p value<5% (Bonferroni method). B–C: MapMan representations of the genes up regulated under infection with prgH − mutant in the ubiquitin-dependent protein degradation pathway. 47 genes are represented. RING and E2 ligases form the second largest cluster. Expression levels 24 h after infection with prgH− or 14028 s wild type was calculated on the base of CATMA array hybridization. Each infection was compared independently to mock treatment (10 mM MgCl2). Differentially expressed genes are presented in Fig. 4. The entire list of differentially expressed genes is presented in Supplementary Data Set S1. D: Enrichment of genes with higher expression levels 24 hours after infection with prgH− mutant compared to the response to infection with 14028 s wild type. GO Term analysis and clustering was done with the help of DAVID Bioinformatic Resources 6.7 at the National Institute of Allergy and Infectious Diseases (NIAID). (TIF) [file pone.0024112.s004.tif]
