# Supplementary material for: Conservation of Salmonella Infection Mechanisms in Plants and Animals
Source: PLoS One. 2011 Sep 6;6(9):e24112. doi: 10.1371/journal.pone.0024112 (PMC3167816; doi:10.1371/journal.pone.0024112)
Supplement: Table S1 — AmiGO enrichment analysis of genes up regulated exclusively during infection with prgH − mutant (http://www.arabidopsis.org/tools/bulk/go/index.jsp). (DOC) [file pone.0024112.s006.doc]

### Supplementary Table S1

AmiGO enrichment analysis of genes up regulated exclusively during infection with *prgH*^-^ mutant (http://www.arabidopsis.org/tools/bulk/go/index.jsp).

| Category: | Nb. of genes* | % in *prgH^-^* set | % in genome | % enrichment |
| --- | --- | --- | --- | --- |
| Response to biotic stress | 43 | 6,8 | 2,1 | 324 |
| Interaction with other organism | 41 | 6,5 | 2,1 | 310 |
| Defense mechanisms | 43 | 6,8 | 2,4 | 283 |
| Response to stress | 104 | 16,4 | 6,5 | 252 |
| Multi-organism interactions | 43 | 6,8 | 2,7 | 252 |
| Response to abiotic stress | 65 | 10,2 | 4,1 | 249 |
| Chemicals | 88 | 13,9 | 6,1 | 228 |
| Small molecules | 65 | 10,2 | 4,5 | 227 |
| Different stimuli | 150 | 23,6 | 11,6 | 203 |
| All *prgH^-^*-specific, differentially expressed genes: 649 | | |  |  |
| * The same gene may be present in different categories, depending on its annotation | | | | |
