# Supplementary material for: Conservation of Salmonella Infection Mechanisms in Plants and Animals
Source: PLoS One. 2011 Sep 6;6(9):e24112. doi: 10.1371/journal.pone.0024112 (PMC3167816; doi:10.1371/journal.pone.0024112)
Supplement: Table S2 — List of genes with the highest difference in expression levels between infections with prgH− mutant and wild-type Salmonella. Table represents log ratios between the mock treatments and the treatment with either prgH− mutant or wild-type Salmonella, and the difference in those two ratios (log prgH-log 14028 s ratio colon) calculated on the base of CATMA microarray analysis. A difference of 1 means that the expression level upon prgH− infection is 2 times higher than upon infection with 14028 s. Value of 1 = log2 1 difference (2× higher expression) between prgH− and 14028 s treatments. (DOC) [file pone.0024112.s007.doc]

### Supplementary Table S2

List of genes with the highest difference in expression levels between infections with *prgH^-^* mutant and wild-type *Salmonella*. Table represents log ratios between the mock treatments and the treatment with either *prgH^-^* mutant or wild-type *Salmonella*, and the difference in those two ratios (log *prgH*-log 14028s ratio colon) calculated on the base of CATMA microarray analysis. A difference of 1 means that the expression level upon *prgH^-^* infection is 2 times higher than upon infection with 14028s.

* Value of 1 = log_2_ 1 difference (2x higher expression) between *prgH^-^* and 14028s treatments.

| **Protein Family** | **AGI** | **log ratio 14028s/mock** | **log ratio** **prgH/mock** | **difference*** |
| --- | --- | --- | --- | --- |
| Protease Inhibitor/LTP | AT2G38870 | 2,63 | 3,95 | **1,32** |
|  | AT4G12500 | 3,33 | 5,18 | **1,85** |
|  | AT3G18280 | 0,10 | 1,49 | **1,39** |
|  | AT4G12470 | 2,17 | 3,52 | **1,35** |
|  | AT4G22470 | 1,69 | 2,91 | **1,21** |
|  | AT4G12490 | 2,86 | 4,04 | **1,18** |
|  | AT1G72060 | 1,07 | 2,55 | **1,48** |
|  | AT2G43510 | 1,07 | 2,55 | **1,54** |
| Extensin | AT1G21310 | -0,01 | 1,33 | **1,34** |
|  | AT1G76930 | 2,85 | 4,26 | **1,41** |
| Glutathion Transferase | AT4G02520 | 1,77 | 3,15 | **1,38** |
|  | AT1G02930 | 1,73 | 2,86 | **1,12** |
| Ca-binding | AT4G27280 | -0,84 | 0,17 | **1,01** |
|  | AT2G46600 | -0,27 | 0,85 | **1,12** |
| Wound-responsive | AT3G10985 | 1,52 | 2,55 | **1,03** |
|  | AT4G28240 | 0,38 | 1,38 | **1,00** |
| LRR | AT1G27190 | 0,72 | 2,08 | **1,36** |
| Chitinase | AT2G43620 | 4,03 | 5,16 | **1,13** |
| PR4 | AT3G04720 | 0,25 | 1,32 | **1,07** |
| Unknown Protein | AT5G66420 | 1,50 | 3,56 | **2,06** |
|  | AT5G42530 | 0,18 | 1,74 | **1,56** |
|  | AT2G25510 | 0,13 | 1,53 | **1,39** |
|  | AT5G22270 | 0,63 | 1,96 | **1,33** |
|  | AT3G15450 | 1,05 | 2,37 | **1,32** |
|  | AT5G35525 | 1,63 | 2,79 | **1,16** |
|  | AT5G44585 | 3,11 | 4,25 | **1,14** |
|  | AT1G25275 | -0,18 | 0,93 | **1,10** |
|  | AT1G65845 | 2,57 | 3,61 | **1,04** |
|  | AT3G02240 | 1,30 | 2,31 | **1,01** |
|  | AT2G46390 | -0,73 | 0,53 | **1,26** |
