# Supplementary material for: Conservation of Salmonella Infection Mechanisms in Plants and Animals
Source: PLoS One. 2011 Sep 6;6(9):e24112. doi: 10.1371/journal.pone.0024112 (PMC3167816; doi:10.1371/journal.pone.0024112)
Supplement: Material and Methods S1 — Detailed description of epithelial cells and mice infection protocols. Description of CATMA-based transcriptome analysis performed in this study with statistical evaluation of the data. (DOC) [file pone.0024112.s008.doc]

## Supplementary Material and Methods

### Epithelial cells assay

*Salmonella* Typhimurium strain 14028s (carrying an amp^R^ plasmid) was grown either *in planta* for two days as described previously , or in LB medium until early logarithmic phase (approximately 5 h at 37 °C). Bacteria were then harvested in PBS with 20 % glycerol and resuspended in PBS. For infection, adequate dilutions of bacteria were made. Human Caco-2 cells (ATCC HTB-37) were grown for two days in DMEM medium supplemented with 2 mM L-glutamine and 10 % fetal calf serum. Antibiotics penicillin (100 µg/mL) and streptomycin (100 µg/mL) were routinely added to the culture medium except 24 h prior to the infection assay. Cells were infected for 1 h at 37°C with different multiplicity of infection (moi) calculated according to cell density counts and serial dilution of bacterial input. Cells were subsequently washed 3 times (5 min each) in PBS supplemented with 100 μg/mL gentamicin and incubated in growth medium (with 10 μg/mL gentamicin) for an additional 2, 4 or 20 h at 37 °C. Cells were lysed in 0.5 % Triton X-100 and adequate dilutions were plated on LB amp^50^ plates in order to monitor the intracellular *Salmonella* population. Three different moi were used for each experiment. Infections were done in duplicates and entire experiment was repeated three times. Calculations were made on the basis of cfu recovered from lysed Caco-2 cells. cfu numbers were normalized to (i) moi number; number of bacteria used for infection divided by the number of epithelial cells or (ii) input population basis, as input we defined bacteria number recovered after 2h post infection. Statistical analysis was performed using the Analysis of Variance (ANOVA) F-test.

### Mice infection assay

Eight-week old female C57BL/6 mice were obtained from Charles River (St. Germain sur l’Arbresle, France) and maintained in our animal facilities on a diet of mouse food and water *ad libitum*, except the day before inoculation where they were deprived of food for 24 h and water for 16 h. Groups of ten mice were each orally inoculated with approximately 5 x 10^5^ cfu of *S.* Typhimurium strain 14028s given in different ways. A first group of animals was inoculated by gavage with 0.2 mL of an inoculum prepared as follows. Bacteria were grown in trypticase soy broth for 24 h at 37 °C with agitation before harvesting and recovering in phosphate-buffered saline (PBS) containing 50 % glycerol. Bacterial cfu were counted after plating serial dilutions on tryptic soy agar plates to determine the bacterial concentration of the inoculum. Aliquots of the inoculum were frozen at -80 °C. On the day of the mice inoculation, one aliquot was diluted in PBS to obtain the desired bacterial concentration. In the second group, each animal was given 0.6 cm^2^ of an uninfected *A. thaliana* leaf to eat; when all mice had eaten their piece of leaf, they were orally inoculated as the first group. In the third group, a 0.6 cm^2^ of a leaf infected with *S.* Typhimurium 14028s was provided as food to each mouse. In the fourth group, mice were inoculated by gavage with a homogenate of *A. thaliana* leaves infected with *S.* Typhimurium 14028 strain. The homogenate was prepared from twenty-five 0.6 cm^2^ pieces of infected leaves. After crushing with a mortar, leaves were immediately resuspended in 5 mL of PBS. In all cases, the number of bacteria given to mice was controlled by plating suitable dilutions on TSA plates. Spleen colonization was estimated at day 3 and 6 (experiment 1) or day 4 (experiment 2) post-inoculation by plating serial dilutions. Results were first analyzed with a non-parametric Kruskal-Wallis test and then all pairs of mice groups were compared with a Dunn’s Multiple Comparisons Test using Instat Sofware (GraphPad). The course of survival was recorded for 21 days following inoculation. Two independent experiments were carried out.

### Construction of *S.* Typhimurium mutants

Mutants of *S.* Typhimurium strain 14028s were obtained using the ambda-Red recombination system . For the *invA* mutant, primers P1-invA (5’TTATATTTGTTTTTATAACATTCACTGACTTGCTATCTGCTATCTCACCGAGTGTAGGCTGGAGCTGCTTC3’) and P2-invA (5’GTGCTGCTTTCTCTACTTAACAGTGCTCGTTTACGACCTGAATTACTGATCATATGAATATCCTCCTTAG3’) were used. For the *ssaJ* mutant, primers P1-ssaJ (5’TCAAAAACGGCGTCTCAGGCAAAAATAGCCGATCAGGATGCCCACTCCTAGTGTAGGCTGGAGCTGCTTC3’) and P2-ssaJ (5’ATGAAGGTTCATCGTATAGTATTTCTTACTGTCCTTACGTTCTTTCTTACCATATGAATATCCTCCTTAG3’) were used. PCR and sequencing of the “scar” sequence and deletion limits were performed to confirm the deletion of *invA* or *ssaJ.*

### Transcriptome analysis

The microarray analysis was performed as described in with modifications. Microarray analysis was carried out at the URGV Plant Genomics (Evry, France), using CATMA arrays containing 24576 gene-specific tags corresponding to 22089 genes from *Arabidopsis* . Total RNA was extracted from 14-day old *Arabidopsis thaliana* seedlings. *Arabidopsis* seedlings were submerged in sterile MS/2 medium without sucrose overnight at 24 °C prior to bacterial treatment. *Salmonella* Typhimurium 14028s or its *prgH^-^* mutant were grown until early log phase and washed with 10 mM MgCl_2_. Infections were performed by inoculation of the MS/2 media with bacteria at OD = 0.1 for 24 h. Two independent biological replicates were produced. For each biological replicate and each treatment, RNA samples were obtained by pooling RNAs from about 20 plants. Total RNA was extracted using QIAGEN RNeasy Plant Kit according to the supplier’s instructions. cRNAs were produced from 2 µg of total RNA from each sample with the Message Amp aRNA kit (Ambion, Austin, TX). Five µg of cRNAs were reverse transcribed in the presence of 300 units of SuperScript II (Invitrogen), cy3-dUTP and cy5-dUTP (NEN, Boston, MA). Samples were combined, purified and concentrated with YM30 Microcon columns (Millipore). Slides were prehybridized for 1 h and hybridized overnight at 42 °C in 25 % formamide. Slides were washed in 2x SSC + 0.1 % SDS for 4 min, 1x SSC for 4 min, 0.2x SSC for 4 min and 0.05x SSC for 1 min and dried. Two hybridizations (dye swaps) were performed for each biological repetition. Each treatment was repeated twice (biological replicate). The arrays were scanned on a GenePix 4000A scanner (Axon Instruments, Foster City, CA) and images were analyzed by GenePix Pro 3.0 (Axon Instruments).

### Statistical Analysis of Microarray Data

Experiments were designed with the statistics group of the URGV Plant Genomics, Evry, France. Normalization and statistical analysis were based on dye swap and biological replication (i.e. four arrays, each containing 24576 GSTs and 384 controls). To determine differentially expressed genes, we performed a paired t-test on the log ratios, assuming that the variance of the log ratios was the same for all genes. Spots displaying extreme variance (too small or too large) were excluded. The raw P-values were adjusted by the Bonferroni method, which controls the Family Wise Error Rate (with a type I error equal to 5 %) in order to keep a strong control of the false positives in a multiple-comparison context. We considered genes with a Bonferroni p-value ≤ 0.05 to be differentially expressed, as described in .

### Supplementary References

1. Schikora A, Carreri A, Charpentier E, Hirt H (2008) The dark side of the salad: *Salmonella* Typhimurium overcomes the innate immune response of Arabidopsis thaliana and shows an endopathogenic lifestyle. PLoS One 3: e2279.

2. Datsenko KA, Wanner BL (2000) One-step inactivation of chromosomal genes in *Escherichia coli* K-12 using PCR products. Proc Natl Acad Sci U S A 97: 6640-6645.

3. Lurin C, Andres C, Aubourg S, Bellaoui M, Bitton F, et al. (2004) Genome-wide analysis of *Arabidopsis* pentatricopeptide repeat proteins reveals their essential role in organelle biogenesis. Plant Cell 16: 2089-2103.

4. Crowe ML, Serizet C, Thareau V, Aubourg S, Rouze P, et al. (2003) CATMA: a complete *Arabidopsis* GST database. Nucleic Acids Res 31: 156-158.

5. Hilson P, Allemeersch J, Altmann T, Aubourg S, Avon A, et al. (2004) Versatile gene-specific sequence tags for *Arabidopsis* functional genomics: transcript profiling and reverse genetics applications. Genome Res 14: 2176-2189.

6. Gagnot S, Tamby JP, Martin-Magniette ML, Bitton F, Taconnat L, et al. (2008) CATdb: a public access to *Arabidopsis* transcriptome data from the URGV-CATMA platform. Nucleic Acids Res 36: D986-990.
